# Aristotelian Practical Wisdom (*Phronesis*) as the Key to Professional Ethics in Teaching

**DOI:** 10.1007/s11245-023-09974-7

**Published:** 2024-02-17

**Authors:** Kristján Kristjánsson

**Affiliations:** Jubilee Centre for Character and Virtues, School of Education, University of Birmingham, Birmingham, B15 2TT UK

**Keywords:** Professional ethics, Teaching, Virtue ethics, *Phronesis*, Ethics education for teacher-trainees

## Abstract

This article is about a virtue ethical approach to the professional ethics of teaching, centred around the ideal of *phronesis* (practical wisdom) in an Aristotelian sense. It is grounded empirically in extensive research conducted at the Jubilee Centre for Character and Virtues into teachers and other UK professionals, and it is grounded theoretically in recent efforts to revive an Aristotelian concept of *phronesis* as excellence in ethical decision-making. The article argues for the need for a virtue-based approach to professional practice, based on time-honoured Aristotelian assumptions and culminating in a conceptually viable construct of *phronesis* as a psycho-moral integrator and adjudicator. After setting some of the historical background in Sect. 1, Sect. 2 charts the most relevant empirical findings. Section 3 introduces a call for *phronesis* as a guide to virtue-based professional ethics: its role, nature, and methods of instruction. Section 4 adds some caveats and concerns about if and how *phronesis* can be cultivated as part of teacher training. Finally, Sect. 5 offers some concluding remarks about the novelty and radicality of the approach on offer in this article.

## Introduction

This article is about professional ethics for teachers and teacher trainees. Although it is grounded empirically in research conducted in my research centre in the UK (see, most recently, Peterson and Arthur 2022), I consider most of the findings and claims made in what follows to be generalisable and scalable internationally, as the classroom contexts of teaching tend to be fairly uniform across the globe, at least in more developed countries. Similarly, while the present focus is on teachers’ professional ethics, many of the reported findings are derived from research with other professionals and professional ethics students also (in medicine, nursing, business, policing, etc.) and are thus potentially applicable to other areas of professional practice and professional ethics education.

Philosophically, this article follows a path already laid by various theorists working within the field of neo-Aristotelian virtue ethics, especially its applied kinds—in its incarnations as ‘character education’ or ‘virtue-based professional ethics’ (Carr 2000; Annas 2011; Kristjánsson 2015). However, it takes Aristotle’s own methodological naturalism seriously by complementing standard philosophical argumentation and conceptual analysis (e.g., of the core construct of *phronesis*) with empirical evidence, thus lending practical traction and ‘street-smart’ credibility to the philosophical arguments. Let us heed Aristotle’s own call here for listening not only to the voices of the ‘wise’ but also of the ‘many’.

After rehearsing some empirical findings about the state of the teaching profession in the UK, I introduce a call for *phronesis* (practical wisdom in an Aristotelian sense) as a guide to virtue-based professional ethics (see also Arthur et al. 2023). I end with a discussion of remaining problems for ethics centred around *phronesis*. However, to provide the relevant background for the following exploration, it is instructive to place it in an historical and theoretical context first.

### Historical and Theoretical Background

Throughout most of the twentieth century, utilitarianism was the dominant moral framework justifying the role of professions in society,^1^ complemented however with a deontological take on the practical ethics of professionals. The way to keep professional agents on the path of appropriate behaviour—and strengthen their public reputation, acknowledged legitimacy, and communal support—was seen to lie in ever-more detailed ethical codes, prescribing correct behaviour, as well as procedures and sanctions to secure such behaviour. Repeated scandals within all the main professions, often exposed by intra-professional whistle-blowers, have shaken the foundations of this conviction. It suffices here to mention the ‘banksters’ responsible for the 2008 financial crisis and the recent revelations of corruption within police forces in countries such as the USA and the UK.^2^ This perception has gone hand in hand with a growing concern among professionals about the loss of the ideal of professional expertise and its replacement with instrumentalist, managerialist orthodoxies that pander to a mistaken confidence in scientific certainties, supplanting personal responsibility and contextual discernment with formalistic accountability and compliance (Schwartz and Sharpe 2010).

As a consequence, focusing attention on the professional *phronesis* of practitioners is now seen by many as a helpful way to rescue professional ethics from the clutches of a stale rule-and-code-based formalism and a culture of mere compliance. This has created a fertile ground for theoretically minded virtue ethicists, operating within the fields of professional ethics, as well as for empirical studies exploring the typical virtues and vices of different professions. In the last 25 years or so, virtue ethics has thus gradually equalled or even surpassed deontology and utilitarianism as the theory of choice within academic professional ethics in areas such as teacher ethics, business ethics, medical ethics, and nursing ethics, although that scholarly interest has not always percolated down to actual professional practice or even to professional ethics education at universities (Kristjánsson 2015, chap. 7).

*Virtue ethics* defines moral rightness according to the effect it has on the agent, in terms of the extent to which it supports the agent to be virtuous and lead a well-rounded flourishing life within a well-ordered community. This ethical theory, as derived originally from Aristotle’s works on ethics and politics, in the West, and Confucian thinkers, in the East, lends itself particularly well to application in professional spheres because of its emphasis on the potential virtuousness of practices and the development of professional expertise—understood as the capacity of *phronesis* or practical wisdom in ethical decision-making—in professional agents such as teachers.^3^ Among other advantages of a virtue ethical approach are its focus on virtuous leadership (e.g., by school principals) and the creation of virtuous communities of people (e.g., teachers in a given field), as well as the strong educational strand that runs through it, in which the development of professional expertise is seen as a life-long journey, intrinsically constitutive of, rather than just instrumentally conducive to, the creation of true professionalism (Arthur et al. 2023).

According to a virtue ethical understanding, professions such as teaching are deemed inherently *ethical occupations* because, more so than other occupations, they place high moral demands on the conduct of workers. Indeed, these ethical and moral demands^4^—which include care, integrity, fairness, and diligence—are often viewed as the *defining feature* of professions, reminding us that professions are ultimately concerned with morally evaluable human actions and interactions. Such demands and standards may also be expected to engender *trust* between professional practitioners and their clients (parents, pupils, etc.), and such trust lies at the heart of professional life. Precisely, the public is entitled to expect professionals to be trustworthy; and trust—which is hard won but easily lost—may be undermined by moral failures and public scandals, as recent examples demonstrate.

## Some Significant Findings About UK Teachers and Other Professionals

Research undertaken by the Jubilee Centre for Character and Virtues between 2012 and 2022 into virtues in UK professions explored the place of virtue in six different professions: law, medicine, teaching, business and finance, nursing, and policing. In each of the profession-specific studies, questionnaires and semi-structured interviews were conducted with first-year and final-year trainees, experienced professionals, and educators. Across all six studies, a total of 4,136 professionals participated.^5^

To report on interviews with teacher trainees and experienced teachers first, what stood out was that the respondents complained that the ‘moral middle’ of the profession gets squeezed out in teacher training, insofar as that training targets ethical issues. What is meant by the term ‘moral middle’ is that because the emphasis is on very *general* principles (e.g., ‘inclusion’, ‘diversity’, in classes for prospective teachers) or very *specific* rules (e.g., about teachers’ dress codes), in addition to formal ethical codes, no time is left to discuss the *middle* sphere: the sphere of actual classroom quandaries and how to deal with those. Consequently, the respondents complained about being torn and pressured, and not being able to ‘act out their real character’ in classrooms, because they had not been given sufficient training in expert ethical decision-making about real-life quandaries. This same complaint is illustrated across all the professions studied when we look at the lack of correspondence between the character strengths and virtues that the participants ascribe to themselves and to the ideal professional (see Table 1).

**Table 1:** Mismatch between self-ascribed and ideal virtues

	Virtue	Difference score
1	*Judgement*	230
2	*Prudence*	230
3	Hope	220
4	Self-regulation	210
5	Bravery	176
6	Leadership	152
7	*Perspective*	143
8	Social intelligence	135
9	Curiosity	112
10	Love of learning	108

Notice that of the 10 virtues with the greatest mismatch, three (the ones italicised) can all potentially be considered ingredients in *phronesis*, as excellence in ethical decision-making.^6^ In other words, aspiring and experienced professionals in the UK do not consider themselves to have had sufficient training in dealing with the nuances of complex decision-making—much of which is uncodifiable, i.e. not possible to capture by pre-determined rules.

**Graph 1:**
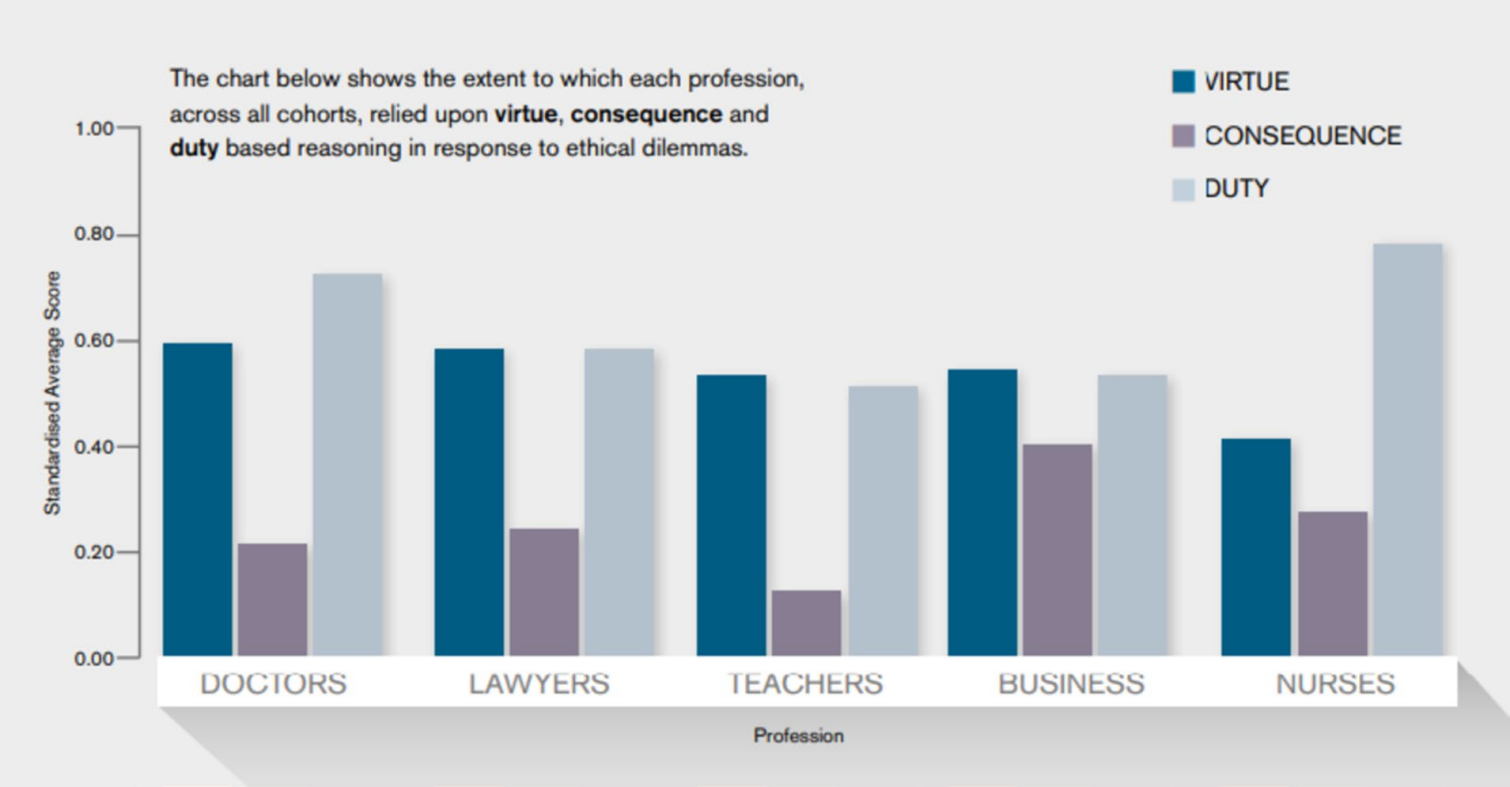
The reliance of different UK professions on virtue ethical, deontological, and consequentialist reasoning strategies

From a virtue ethical perspective, Graph 1, however, presents a relatively positive image of teachers’ decision-making strategies compared to many other UK professions. This graph shows the extent to which participants draw on virtue ethical, deontological (rule-based), and consequentialist reasoning strategies when presented with work-place dilemmas.

**Graph 2:**
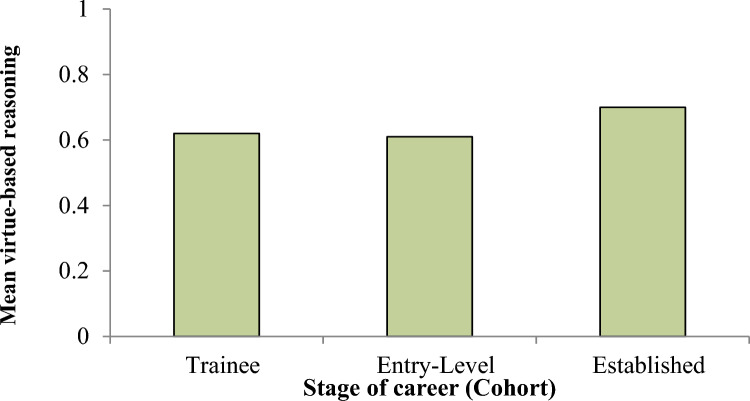
Reliance on virtue ethical reasoning among different cohorts of UK Professional students and practitioners

So, teachers seem to be the ones least reliant on mere formal duties when making difficult decisions. However, like in most other UK professions, the confidence in one’s own compass takes a dip from the first-year of undergraduate study to the final year (perhaps because of the strong focus in professional ethics classes on formal codes), although it picks up again after some time working in the field (see Graph 2).

**Graph 3:**
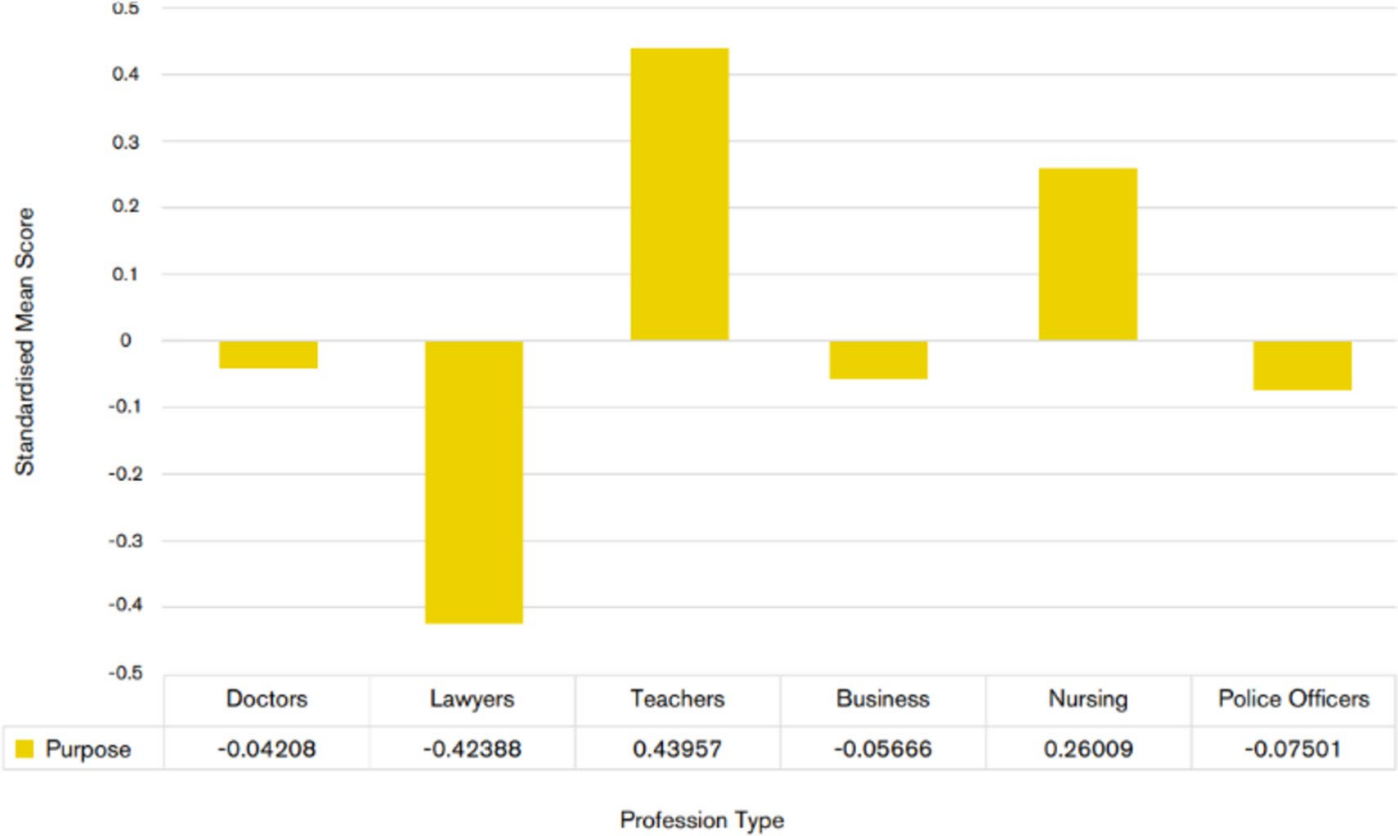
Standardised mean differences in professional purpose across six UK professions

Let me next report on a finding that, again, shows the teaching profession in the UK in a positive light. We explored experienced professionals’ sense of *professional purpose*, understood as their sense of the worth of their professional activities and their contribution to the greater good, in a context considered worthy of operating within. Despite vocal complaints from teachers in the UK about being held back professionally by various factors, they scored quite high on professional purpose (see Graph 3).

Despite this comparatively high score overall, however, interviews with individual teachers revealed various reasons that threaten to undermine their sense of purpose, such as:

- Perceived failures to be able to act out one’s personal moral character traits in the given professional context.
- A general sense of one’s professional context not being conducive to professional development.
- A sense of an overbearing and inflexible managerial structure that does not allow for individual professional *phronesis*.
- Feelings of inadequacy in negotiating dilemmatic situations in the workplace.
- A sense of belonging to a profession that is not experienced as worthy by the general public or by employers.

The final empirical finding to be reported upon in this section is about the connection between the perceived possession of certain moral and intellectual virtues and a sense of professional purpose. Participants^7^ were asked to rank their top six character qualities from a list of 24, and to also rate how strongly they agreed or disagreed with six statements on their feelings of professional purpose using a 5-point scale. From the ranked character qualities, four distinct character-judgement profiles were identified; *low group* (those who valued moral character and intellectual judgement below the sample average), *judgement-only group* (those who valued intellectual judgement above the sample average), *character-only group* (valued moral character above the sample average), and *high group* (valued moral character and intellectual judgement concurrently).

**Graph 4:**
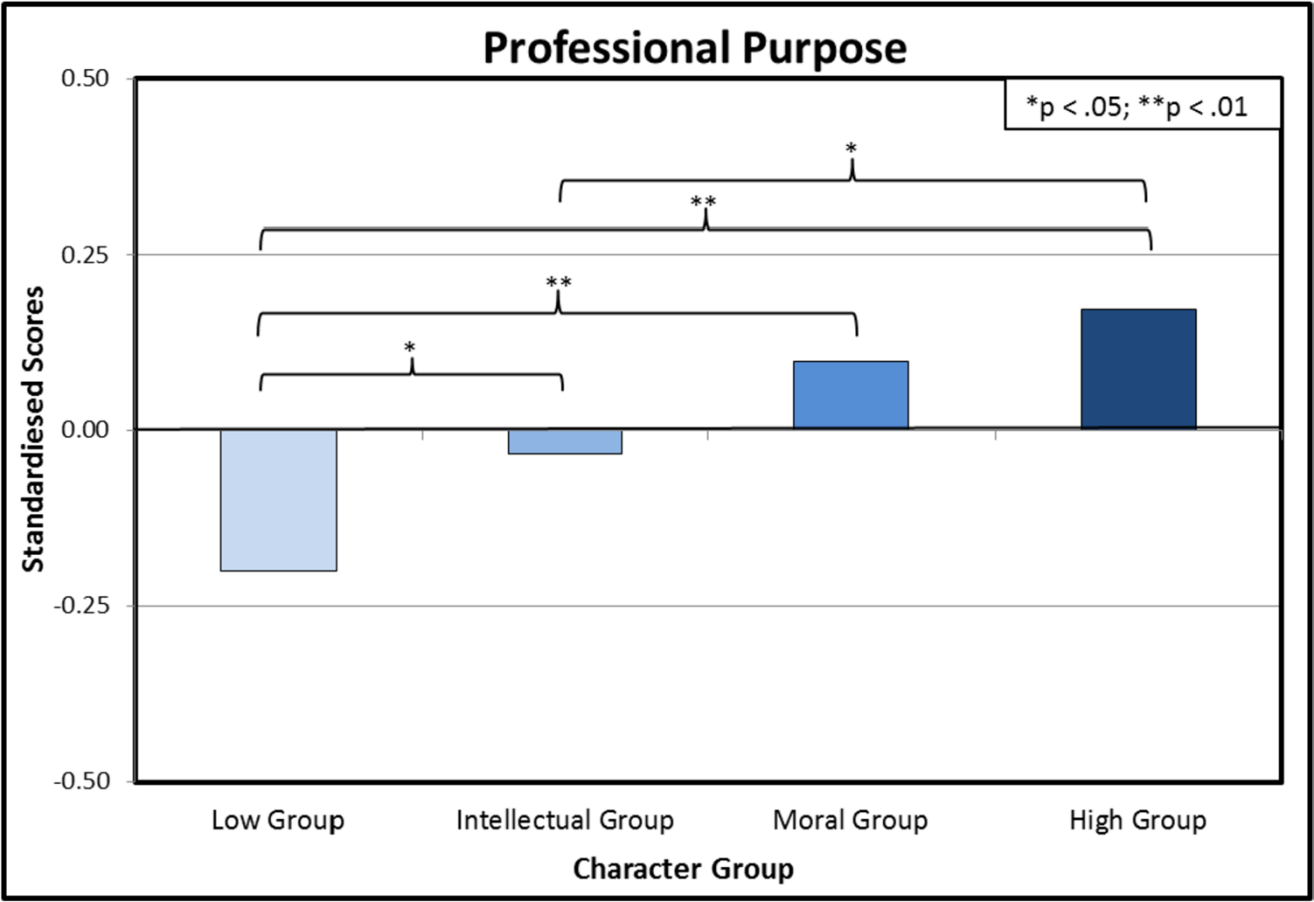
Differences in professional purpose depending on self-ascribed virtues

Graph 4 shows that those who ascribe to themselves a combination of moral and intellectual virtues have the strongest sense of professional purpose. This finding (albeit correlational rather than causal) is highly relevant to the argument to be made in the remainder of this article about the value of *phronesis* for the professional ethics of teachers, because *phronesis* (as explained in Section III) includes a combination of moral virtues and an intellectual metacognitive capacity to integrate those virtues and reach a well-deliberated-upon judgement in cases where there seems to be a discordance between the motivations behind different moral virtues.

## The Role, Nature, and Teaching of *Phronesis*

The concept of *phronesis* (practical wisdom), in general, and professional *phronesis*, in particular, is nothing less than the key concept holding together the approach of virtue-based professional ethics represented in this article. Indeed, the biggest growth industry in *phronesis* research in the last couple of decades has not been within philosophy, psychology, or even moral/character education, but rather within *professional ethics*: the ethics of medicine, teaching, nursing, business, social work, policing, the military, and so forth. Schwartz and Sharpe’s (2010) popular book on practical wisdom, which seems to have spurred some of the recent interest in *phronesis*, highlights particularly the use (or absence) of *phronesis* within professional practice. I strongly recommend this book as a preliminary reading to any aspiring students of *phronesis*, and especially so within the ethics of teaching. It neatly sets the background of the motivation to reclaim *phronesis* as an ideal, in an age of ever tighter and better regulated (but essentially fallible) audit cultures, in which professional wisdom has increasingly been de-skilled and replaced with rules, codes, and incentives, as explained earlier. The book is a goldmine of examples, many of which are derived from professional practice, of why the carrots-and-sticks method does not work and why it is essentially anti-professional.

### What Is *Phronesis*?

Generally speaking, *phronesis*, as defined by Aristotle (1985), is the intellectual meta-virtue that helps a moral agent to integrate and adjudicate upon the (sometimes) conflicting messaging coming from the different moral, civic, and performance virtues.^8^ In a sense, then, *phronesis*, is the conductor of the whole ‘virtue orchestra’. Reimagining the ideal of professional *phronesis* in teaching means re-equipping teachers with the capacities and responsibilities to make excellent ethical decisions themselves, building on their moral/civic virtues and their insights into situational complexities—which can never be replaced with codified formulas. According to recent neo-Aristotelian analyses, *phronesis* encompasses four different functions (Kristjánsson et al. 2021a; Kristjánsson and Fowers 2024)^9^:

#### Constitutive Function

*Phronesis* involves the cognitive ability to perceive the ethically salient aspects of a situation and to appreciate these as calling for specific kinds of responses. This ability can be cultivated in teacher trainees as the capacity to ‘read’ a situation by seeing what is most important or central.

#### Blueprint Function

The integrative work of *phronesis* operates in conjunction with the teacher’s overall understanding of the kinds of things that matter: the teacher’s own ethical identity, aims, and aspirations, her understanding of what it takes to live and act well and her need to live up to the standards that shape and are shaped by her understanding and experience of her professional life. This amounts to a blueprint of professional flourishing.

#### Emotional Regulative Function

Teachers foster their emotional wellbeing through *phronesis* by bringing their emotional responses into line with their understandings of the ethically salient aspects of their situation, their judgement, and their recognition of what is at stake in the moment. For example, a teacher might recognise that her appraisal of the situation is problematic, giving rise to an emotional response that is inappropriate to the situation. The emotional regulative function can then help her adjust her appraisal and emotion by, for instance, giving herself an inner ‘talking to’.

#### Integrative/Adjudicative Function

Through *phronesis,* a teacher integrates different components of a good life, via a process of checks and balances, especially in circumstances where different ethically salient considerations, or different kinds of virtues or values, appear to be in conflict. In some cases, integration may call for a ‘blended’ or ‘synchronised’ virtuous response, such as being compassionately honest or honestly compassionate; in other cases, a virtue may have to be put on hold completely in a given situation in light of the overriding requirement of a conflicting virtue. Therefore, this function allows the person to engage in the adjudication of moral matters when conflicting desiderata arise.

**Fig. 1:**
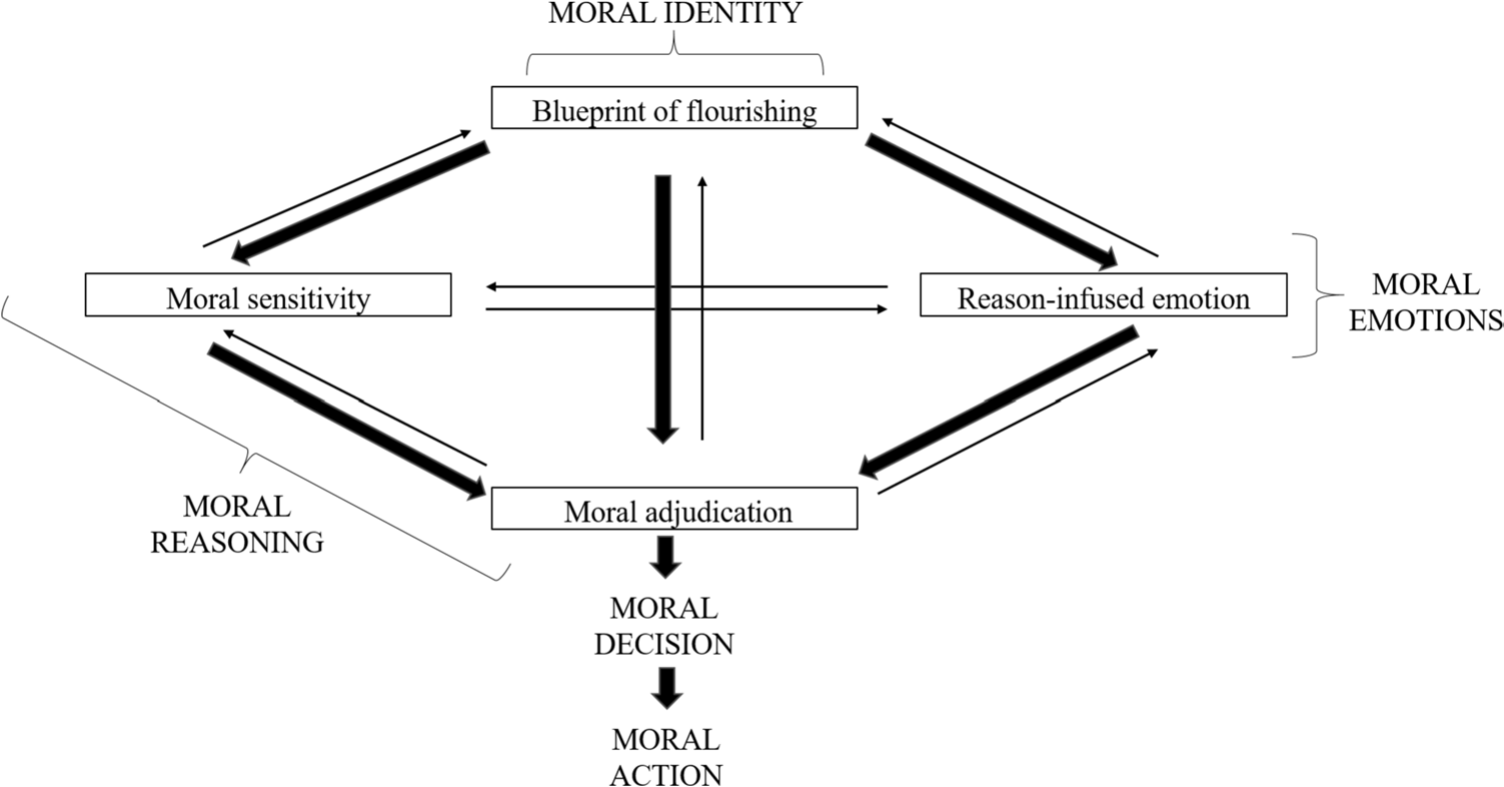
A Neo-Aristotelian model of wise (*phronetic*) ethical decision-making

Figure 1 illustrates the overall conceptualisation of *phronesis* (Kristjánsson et al. 2021a).

Without *phronesis*, the different aspects of a teacher’s virtuous make-up will fail to become integrated. A lack of attention to *phronesis* in teaching practice and teacher training thus amounts to an act of de-professionalisation.

### How Can We Educate *Phronesis*?

Because Aristotle himself was fairly reticent about the nuts and bolts of *phronesis* education, we must rely on various educated guesses and hypotheses (Kristjánsson 2021). What seems clear is that *phronesis*-guided ethics education needs to begin with the ‘constitutive function’ (also known as moral sensitivity): the ability to identify the ethical issues at stake. Teacher trainees need to be presented with workplace dilemmas and asked to analyse them, as well as the available action options. A lot of this initial educative work simply involves *virtue literacy*: the ability to spot virtues, name them, and apply them to one’s own domains of experience.

Much of professional ethics will be *caught* from the work environment and organisational culture through ‘osmosis’. A non-virtue-friendly ethos in schools, for instance one steeped in rules and regulations but inimical to individual reflection, can thus hinder the development and execution of *phronesis*. This claim has a wider application: *phronesis* will not be caught from a learning environment that is inimical to virtue development in general. As Russell notes (2021, 17), the difficulties in learning *phronesis* are not only caused by its ‘high intrinsic load’ (the need to synthesise cognitively different values and virtues) but also by a ‘high extrinsic load’ (environments typically full of misleading feedback, bad advice, and false friends^10^). Whatever the quality of teaching materials on offer, for example in the form of a well-designed CPD course for teachers on virtue-based professional ethics,^11^ no significant learning will take place unless the workplace culture is conducive to such learning.

*Role-model education* is a staple of early-years character development. However, being typically fairly uncritical at that stage, this method comes with a known plethora of problems: moral inertia, moral over-stretching, and hero-worship. Will those problems disappear if role-model education is introduced as part of a more critical and reflective programme of *phronesis-*guided professional ethics education? There is reason for caution here. The situations in which role models exhibit practical wisdom are often highly specific and far removed from the experiential worlds of the students. Moreover, lessons from stories about role models are often pitched at too high a level of generality, not taking account of the contextual/perspectival nature of practical wisdom. For example, even King Solomon was unfortunately not wise across the board. The best role models for professional ethics education are attainable and relatable ones (Han et al. 2017; cf. Henderson 2024). Indeed, the ideal moral exemplars are not literary figures but people who are already close to the moral learner, such as university mentors within the relevant profession or *phronetic* workplace colleagues—namely, exemplary fellow teachers—who can serve as models for emulation.

When it comes to methods of how (professional) *phronesis* can be *taught* in such a way that it will be subsequently *sought* and pursued by professional ethics students, the plot thickens. A survey of the relevant background literature suggests that the diffusion characteristic of extant *phronesis*-relevant interventions lies in the fact that most such interventions do not take any distinct model of *phronesis* as their starting point, and that they almost invariably work (implicitly) on just one, or maximum two, components of *phronesis*, rather than the virtue as a whole. For example, an interesting project on the development of social reasoning, based on dialogical and collaborative methods (Lin et al. 2019), might be good at developing the constitutive and integrative functions of *phronesis*, but it has little to do with the blueprint function or the emotional regulative one. A host of interventions to develop ‘emotional intelligence’ via ‘social and emotional learning’ exist, but they usually do not target other aspects of *phronesis*, nor indeed see themselves as having anything to do with morally relevant practical wisdom as such. Quite a lot is known about how to build a sense of purpose and moral identity in young people (Damon 2008), but less is known about how such teaching can interact with work on the other components of *phronesis*, or how it contributes to a sense of professional purpose among teachers.

Every cloud has a silver lining, however. A lot of educational research exists, *under other designations*, that actually appears to be about the cultivation of what Aristotle called ‘*phronesis*’ (or at least crucial components of it), either indirectly or directly. Notice here research about metacognitions, post-formal thinking, self-reflection, social reasoning, professional expertise, tacit knowledge, and various other related topics (cf. Kallio 2020). The trick, then, is not to reinvent the wheel but try to build on what other researchers have done in overlapping areas (cf. Sternberg 2001). In other words, new interventions will not need to be constructed de novo; the key will lie in combining them together correctly under the guidance of holistic models like the *phronesis*-model presented earlier. There is every reason to believe that light will emerge at the end of this tunnel, as 96% of the wisdom researchers that Grossmann et al. (2020, 117) surveyed believed that practical wisdom is malleable in principle.

We in my research centre have not yet had a chance to create a *phronesis* intervention for teacher trainees or experienced teacher built on the above model of *phronesis*. As an example of what teaching professional *phronesis* can look like in practice, however, consider a recent Jubilee Centre intervention to teach *phronesis* to police-science students. Only having four classes to play with, it was decided to devote those mostly to a deep discussion of topical police dilemmas. The choice of those dilemmas was tricky—they would have to be *relevant*, *realistic,* and *relatable* (Han et al. 2017). However, the Centre was aided here by the work of an Expert Panel who had already helped create dilemmas for a previous study (Kristjánsson et al. 2021b). After introducing the dilemmas, through a guided discussion, the students were asked to discuss and reach a conclusion about various questions, including: (1) Which virtues or values are competing and steering the police officer in different directions? (2) What are the pros and cons of each action option? (3) Is the police officer experiencing strong emotions prior to the decision? (4) If so, what are those emotions? (5) What should the police officer do, in your view? At the close of the intervention, the students were asked to relate their answers to the police Code of Ethics. A post-test with a *phronesis* measure (Darnell et al. 2022,12) was then administered to gauge whether progress had been made during the intervention in *phronetic* decision-making (compared to a pre-test with the same measure), with respect to one or more of the components/functions of *phronesis*.

The intervention, sketched above, was no rocket science. The aim was, somewhat obviously, to help students develop the different components of *phronesis*, by taking them through some of the considerations that motivate and (ideally) strengthen each component. The method of teaching was a guided discussion about relevant dilemmas: a method that has a long history in approaches to moral education. To couch the rationale of the intervention in a slightly more academic educational language, it was set within what was deemed to be the police-science students’ ‘zone of proximal development’ (ZPD) as *phronesis* learners. In line with Vygotsky (1978), that zone sits between two other zones, of (1) what students can learn by themselves without going through the actual future experiences and (3) what students will have learned after going through the actual future experiences. The ZPD marks the in-between zone of (2) what students can learn prior to the actual experiences through ‘scaffolded teaching’ by a skilled tutor. The key to a successful professional ethics education, following an Aristotelian virtue ethical approach, is to define zone (2) with sufficient specificity and work on it through guided practice.

The police intervention described here could of course be replicated for teacher trainees by replacing the police-work dilemmas with dilemmas from teachers’ classroom practice. Apart from a changed content, the same methodology could be used. However, our experience of the police intervention indicates that four classes are not sufficient to ground a deep understanding of *phronesis*, especially for students who have no prior knowledge of the relevant philosophy or psychology. Indeed, it is not unreasonable to suppose that a virtue ethical *phronesis*-guided approach to the professional ethics of teachers requires a sustained focus throughout the whole period of teacher training for it to be truly ingrained in the moral character of the students. It cannot just be an ‘add-on’ to one module in their studies.

## Remaining Problems

Some theorists consider Aristotle to be overly demanding about the intellectual nature of ethical decision-making. For Aristotle, doing the right thing does not have any moral value unless it is done for the right (*phronetic*) reasons and from the right motives (Aristotle 1985, 40 [1105a30–34]). Mere prosociality (good social outcome) does not seem to matter at all. Are parents and teachers not concerned with teachers acting in the right way, without necessarily caring about the underlying motives? Yes, of course everyone wants teachers to do and say the right things. However, the virtue ethical point would be that we cannot count on teachers as consistent moral agents if their actions are merely extrinsically motivated. We want them to develop an intellectually guided (i.e., *phronetic*) moral character that motivates them intrinsically and reliably in every domain of ethical decision-making in the school and elsewhere.

Yet educating the *phronetic* teacher is a tall order with many problems attached to it. Here is the first problem. Teaching is—along with professions such as medicine, nursing, and policing—a *burdened profession* in the sense of one in which practitioners are likely to encounter various psychologically charged, and even life-changing, situations that are impossible to explain to students in sufficient depth before they encounter them. These are also professions with a high rate of burn-out, perhaps because of various factors that gradually seem to sap the practitioners’ original moral purpose in entering them (Arthur et al. 2021). However, not only is it impossible to explain many of these sources of burden to students until they experience them themselves in their teaching practice, we do not even know what are going to be the main dilemmas facing teachers in 20–30 years from now.

Another and related problem is that any realistic dilemmas presented to teacher trainees will involve experiences that are in a fundamental sense *embodied*. I am not using the term here in any obscure philosophical sense, but simply as referring to the fact that the experiential context involves physical processes and emotions as well as mental reflection.^13^ To give an analogy, it is almost impossible to explain to a young child what sexual jealousy feels like and how those feelings will affect moral decision-making once the relevant adolescent hormones have kicked in. The child will perhaps know what sibling jealousy feels like, and analogies can be drawn with those experiences; but they are not the same as the experiences of sexual jealousy. Similarly here, some of the dilemmas presented to teacher-training students will by necessity involve situations that are bound to elicit strong physical and emotional reactions—but ones which cannot be known ‘in one’s skin’ prior to the event.

In short, we are dealing here with a ‘zone of proximal development’ that is severely circumscribed by the fact that the situations for which the students are being prepared are experientially conditioned and embodied. All that can be achieved within the ZPD is an *intellectual* exercise that may, at best, stimulate certain discrete components of *phronesis* but can only partially account for the context in which the eventual decision will be set.^14^ If we venture further than that, in attempting to expand the ZPD, two perils await us. One is *developmental naivety*, in which complex experiences are reduced to an intellectual exercise in an attempt to articulate something that is inarticulable out of context—possibly inducing the infamous Dunning-Kruger effect.^15^ The second is *paternalism*, in which we cavalierly ignore the students’ need to engage in their own ‘experiments in living’ prior to becoming capable of making autonomous moral decisions, be those professional or personal.

That said, the temptation is very strong to expand the ZPD, especially if the tutors have gone through some of those experiences themselves and perhaps made mistakes that they want to pre-empt in students. The educational dilemma created here is a well-known one, with implications far beyond any interventions to cultivate Aristotelian *phronesis*. On the one hand, we may have tutors who know in their own skin what typically ‘happens to the heart’ in the relevant profession; on the other hand, we have budding professionals who have not gone through those experiences and are full of idealism about their future work. The tutors do not want to curb the students’ idealism; but they also want to convey to them a sense of the challenges ahead (see further in Kristjánsson 2022b).

Even the two most vocal champions of *phronesis* as part of professional ethics education claim that it ‘is not something that can be taught’ (Schwartz and Sharpe 2010, 271)—although they probably understand the term ‘teaching’ more narrowly in this context than Aristotle did. While I would not go as far as Schwartz and Sharpe, it is worth reminding readers of the well-known Chinese fable of the farmer who impatiently tried to pull up his rice shoots to make then grow faster, as a result of which they lost their rootedness and withered away. Young teacher trainees, for instance, need to be fed a diet that does not exhaust their capacities for digestion—which is not the same as saying that they should not be provided with an intellectual initiation into some of the tough and discretionary choices that await them and with a stark warning that no rule book will relieve them of the responsibility for making those choices themselves.

I want to mention finally one problem that is *institutional* rather than educational in a more narrow sense. The strict top-down control of teaching in many countries has seriously limited the scope for *phronetic* decision-making in the classroom. Harðarson (2019) therefore wonders whether it is fair to expose teacher trainees to the ideal of *phronesis* if they are then debarred from using this mode of thinking when they enter the workplace. Similarly, Jameel (2022) despairs about the bureaucratic culture that has eroded the foundations of family medicine as a *phronetic* practice. The point made here is a highly relevant, if a deflationary, one. If schools are not organised in such a way that teachers’ autonomy and critical decision-making is valued and systematically relied upon, why should we foreground this in teacher training? This question shows that decisions about the content of professional ethics education for aspiring teachers cannot be seen solely as decisions about what is to be taught in an individual module or two; these are decisions that have to do with the overall aims of schooling and the role that we want teachers to play in the schools of the future. Professional ethics in teaching is, therefore, not a siloed subject; it must be pursued in conjunction with much deeper and more far-reaching questions about the aims of education and schooling in general.

## Concluding Remarks

Recent empirical literature is full of examples, from all over the world, of how badly teachers deem themselves prepared for tackling life’s biggest questions in the classroom. They complain about a lack of attention to normative issues in teacher training, and about their own lack of moral language and moral identity. As Chris Higgins correctly observes, ‘restoring to its central place the flourishing of the practitioner is the first step in constructing a virtue ethics of teaching’ (Higgins 2011, 10). Before teachers can help students answer adequately the question of what kind of persons they want to become, in order to fulfil their potential, the teachers themselves need more extensive training in how to ask and answer such questions about themselves, both at the professional and personal levels (see also Carr 2000).

I have argued in this article, both on grounds of the empirical evidence presented in Section II and the theoretical considerations in Section III, that the best way of preparing teaching students for the ethical quandaries that meet them in schools and classroom is through the cultivation of *phronesis* and that this construct should form the lynchpin of a virtue ethical approach to the professional ethics of teaching. This does not mean that we can get rid of all formal ethical codes. Those can helpfully set *minimal expectations* and pinpoint aspects of teacher–student interactions that are *categorically prohibited* (such as sexual relations or acts of racial/ethnic discrimination). However, professional ethics for teachers that is based solely on deontological principles and codified rules—motivated by carrots and sticks—constitutes a very thin gruel, which gives insufficient ethical nutrition and can even cause ethical indigestion (by being anti-professional and depriving teachers of a sense of professional purpose).

A virtue ethical approach to professional ethics, centred around *phronesis*, signifies quite a radical new agenda. Such a change of compass within professional ethics—that is, the incarnation of virtue ethics in professional fields—is in many ways more radical than the incarnation of virtue ethics as character education in primary and secondary schools. In most decent schools, teachers have always acted as character educators. Character education just makes those efforts more conscious and systematic. However, in professional domains, the move towards *phronesis*-guided virtue ethics signals a radical turn away from the status quo, which—according to most of the professionals who we in the Jubilee Centre interviewed between 2012 and 2022—involved no engagement whatsoever with moral character in professional ethics classes. They were all about audits, codes, and compliance. Hence, we are really targeting something new and ground-breaking here. Nevertheless, for Western teachers it establishes a bond back to ancient Greek ideas about virtue-based ethical competence, and for Eastern teachers, it potentially forges links with an ancient Confucian tradition.

In today’s world, we need teachers who act as *ethical stewards*, developing their own moral character and the character of their students at the same time through ethical classroom practice.

## Footnotes

1 For example, until recently, ‘human capital theory’ was the official doctrine of OECD about the aims of education, in an international context. According to this theory, teaching aims at increasing societal (GNP) and individual (income-potential) prosperity. This is a utilitarian way of understanding education’s aims. More recently, however, OECD has been leaning more in a virtue ethical direction, with a focus on flourishing (including the development of character virtues) as the aim of education (Stevenson 2022).

2 Although the teaching profession has not been as hard hit by recent scandals, the Me-Too movement unearthed various cases of sexual misdemeanours exhibited by teachers and professors towards (mainly female) students. Notice here also that a number of professions have been given ethical responsibilities that go beyond the self-improvement of the relevant professionals, to include the improvement of others as well. Teaching is an example of a profession that has a ‘Janus-faced’ (facing both inwards and outwards) ethical mission, for insofar as moral or character education is seen as an aim of schooling, teachers have the role of helping students cultivate moral, civic, intellectual, and performance virtues. However, taking on this ‘Janus-faced’ mission places additional expectations on the professionals in question, which is the reason why the general public are particularly sensitive to professional misconduct witnessed among teachers.

3 On the relationship between expertise, professionalism, and *phronesis*, see e.g. Carr (2000); De Caro et al. (2021); Stichter (2021). Notice, however, that these authors do not endorse the same conceptual model of *phronesis* as introduced below.

4 While the terms ‘moral’ and ‘ethical’ are often used interchangeably in the literature, ‘ethical’ is sometimes used to capture that subset of the ‘moral’ which has to do with domain-specific (e.g. profession-specific) norms and aspirations. Notice that this article is about the ‘ethical teacher,’ in general, rather than the about the ‘teacher of ethics’ or morality/character to students, in particular (see also Campbell 2003).

5 The findings presented in the current section are derived from various Jubilee Centre reports available for downloads here: https://www.jubileecentre.ac.uk (under Virtues in the Professions). The most up-to-date publication about teachers in particular is Peterson and Arthur (2022). For a synthesis of findings, see Arthur et al. (2021).

6 McGrath (2019) argues that these three virtues can substitute *phronesis* in the Values-in-Action classificatory system of positive psychological character strengths and virtues. While I very much doubt that this is the case, there is no space to critique that argument here. Let me simply point out that it is not entirely clear whether judgement, prudence, and perspective (individually or collectively) entail action. However, it is clear in the Aristotelian system that *phronesis qua* excellence in decision-making is not only about excellence in deliberation/reflection but also excellence in action. Furthermore, there is an apparent overlap between *phronesis* and some other virtues in the positive psychological system, such as self-regulation (which also figures in Table 1), given what I say about emotional regulation as a component of *phronesis* later.

7 In this case, we looked at participants from teaching, medicine, nursing, and business (Arthur et al. 2021).

8 I rely here on a distinction between four kinds of virtues common among current neo-Aristotelians pursuing character education or virtue-based professional ethics (e.g. Arthur et al. 2023). Aristotle himself would not have considered the performance virtues real virtues, but rather just skills (*techné*), because they lack intrinsic value. Similarly, he would have called the moral virtues ‘characterological’ (because he had no concept of ‘the moral’ in ancient Greek) and his name for the civic virtues was ‘political virtues’. See further in Kristjánsson and Flowers (2024).

9 Notably, not all neo-Aristotelians carve up the functions of *phronesis* in exactly the same way (see further in Kristjánsson and Flowers 2024, chap. 2). Moreover, there are non-Aristotelian accounts of *phronesis* competing with the Aristotelian ones (most prominently De Caro et al. 2021). While competing in some ways, all these accounts would probably agree with the broad definition of *phronesis* as ‘excellence in ethical decision-making’. For present purposes, the below four-componential model suffices as one standard representation.

10 Although the focus on a virtue-conducive work ethos is school is most often on school policies and school management, it is also important to have supportive colleagues who help each other grow in virtue. Notice here Aristotle’s emphasis on the development of good character through interactions with ‘equal friends’ (i.e., peers) who acts as our critical interlocutors (Kristjánsson 2022a). For examples of how workplace jealousies in schools can, for instance, undermine professional development, see Chen and Kristjánsson (2011).

11 The Jubilee Centre has recently created a free CPD course of this kind: https://www.jubileecentre.ac.uk/2997/projects/virtues-in-the-professions/online-cpd-professional-ethics

12 While satisfactorily validated, this original measure took too long to administer and was unwieldy to score. Subsequently, a shorter and more thoroughly validated measure of *phronesis* has been produced (Kristjánsson et al. 2023).

13 Cf. here Hargreaves’ well-known writings about teaching as ‘emotional labour’ (e.g., Hargreaves 1998).

14 Unsurprisingly, Ardelt (2020) found that that a wisdom intervention that targeted ‘the whole person’ had a greater effect than more context-and-discipline-specific teaching. At the same time, Grossmann’s (2017) research indicates that teaching about how wisdom exemplars react to dilemmas is most beneficial to students if those examples are situated within specific contexts that the exemplars encountered and mastered. Those findings are not incompatible. If practical wisdom is a multi-component construct, it is likely that a broad approach to enhancing it will be the most effective *pedagogical strategy*. On the other hand, the *content* of the stories used to hone the different components may need to be highly situation-specific for it to resonate with the students.

15 The Dunning-Kruger effect is a cognitive bias through which people with limited knowledge or competence in a given intellectual or social domain greatly overestimate their own knowledge or competence in that domain.

## References

[CR1] Annas J (2011). Intelligent virtue.

[CR2] Ardelt M (2020). Can wisdom and psychosocial growth be learned in university courses?. J Moral Educ.

[CR3] Aristotle (1985). Nicomachean ethics, trans. T. Irwin.

[CR4] Arthur J, Earl SR, Thompson AP, Ward JW (2021). The value of character-based judgement in the professional domain. J Bus Ethics.

[CR5] Arthur, J., Kristjánsson, K., Thompson, A.P., and Fazel, A. (2023). The Jubilee Centre framework for virtue-based professional practice. https://www.jubileecentre.ac.uk/?project=framework-for-virtue-based-professional-ethics

[CR6] Campbell E (2003). The ethical teacher.

[CR7] Carr D (2000). Professionalism and ethics in teaching.

[CR8] Chen Y-H, Kristjánsson K (2011). Private feelings, public expressions: professional jealousy and the moral practice of teaching. J Moral Educ.

[CR9] Damon W (2008). The path to purpose: how young people find their calling in life.

[CR10] Darnell C, Fowers B, Kristjánsson K (2022). A multifunction approach to assessing Aristotelian *phronesis* (practical wisdom). Personality Individ Differ.

[CR11] De Caro M, Marraffa M, Vaccarezza MS, De Caro M, Vaccarezza MS (2021). The priority of *phronesis*: how to rescue virtue theory from its critics. Practical wisdom: philosophical and psychological perspectives.

[CR12] Grossmann I (2017). Wisdom and how to cultivate it. Eur Psychol.

[CR13] Grossmann I, Weststrate NM, Ardelt M, Brienza JP, Dong M, Ferrari M, Fournier MA, Hu CS, Nusbaum HC, Vervaeke J (2020). The science of wisdom in a polarized world: knowns and unknowns. Psychol Inq.

[CR14] Han H, Kim J, Jeong C, Cohen GL (2017). Attainable and relevant moral exemplars are more effective than extraordinary exemplars in promoting voluntary service engagement. Front Psychol.

[CR15] Harðarson A (2019). Aristotle’s conception of practical wisdom and what it means for moral education in schools. Educ Philos Theory.

[CR16] Hargreaves A (1998). The emotional practice of teaching. Teach Teach Educ.

[CR17] Henderson E (2024). The educational salience of emulation as a moral virtue. J Moral Educ, in Press.

[CR18] Higgins C (2011). The good life of teaching: an ethics of professional practice.

[CR19] Jameel SY (2022) Enacted *phronesis* in general practitioners. Unpublished PhD thesis. University of Birmingham. https://etheses.bham.ac.uk/id/eprint/12197/

[CR20] Kallio EK, Kallio EK (2020). From multiperspective to contextual integrative thinking in adulthood: considerations on theorisation of adult thinking and its place as a component of wisdom. Development of adult thinking: interdisciplinary perspectives on cognitive development and adult thinking.

[CR21] Kristjánsson K (2015). Aristotelian character education.

[CR22] Kristjánsson K (2021). Twenty-two testable hypotheses about *phronesis*. Br Edu Res J.

[CR23] Kristjánsson K (2022). Friendship for virtue.

[CR24] Kristjánsson K (2022). Teaching *phronesis* to aspiring police officers: some preliminary philosophical, developmental and pedagogical reflections. Int J Ethics Educ.

[CR25] Kristjánsson K, Fowers BJ (2024). *Phronesis*: retriving practical wisdom in psychology, philosophy, and education.

[CR26] Kristjánsson K, Fowers BJ, Darnell C, Pollard D (2021). *Phronesis* (practical wisdom) as a type of contextual integrative thinking. Rev Gen Psychol.

[CR27] Kristjánsson K, Thompson A, Maile A (2021b) Character virtues in policing: research report. https://www.jubileecentre.ac.uk/wp-content/uploads/2023/07/CharacterVirtuesinPolicing_ResearchReport.pdf

[CR28] Kristjánsson K, McLoughlin S, Thoma S (2023) *Phronesis*: developing and validating a short measure of practical wisdom. Research report. https://www.jubileecentre.ac.uk/wp-content/uploads/2023/12/Phronesis_DevelopingAndValidatingAShortMeasureOfPracticalWisdom-_Final-1.pdf

[CR29] Lin T-J, Ha SY, Li W-T, Chiu Y-J, Hong Y-R, Tsai CC (2019). Effects of collaborative small-group discussions on early adolescents’ social reasoning. Read Writ.

[CR30] McGrath RE (2019). Refining our understanding of the VIA classification: reflection on papers by Han, Miller, and Snow. J Positive Psychol.

[CR31] Peterson A, Arthur J (2022). Ethics and the good teacher.

[CR32] Russell DC, De Caro M, Vaccarezza MS (2021). The reciprocity of the virtues. Practical wisdom: philosophical and psychological perspectives.

[CR33] Schwartz B, Sharpe K (2010). Practical wisdom: the right way to do the right thing.

[CR34] Sternberg RJ (2001). Why schools should teach for wisdom: the balance theory of wisdom in educational settings. Educational Psychologist.

[CR35] Stevenson M (2022) Education for human flourishing. Centre for Strategic Education. https://drive.google.com/file/d/1LKxCvKbk6wFh2xQffxv5hIVCjy8STVKt/view

[CR36] Stichter M, De Caro M, Vaccarezza MS (2021). Differentiating the skills of practical wisdom. Practical wisdom: philosophical and psychological perspectives.

[CR37] Vygotsky LS (1978). Mind in society: the development of higher psychological processes.

